# Fear of the human ‘super predator’ in native marsupials and introduced deer in Australia

**DOI:** 10.1098/rspb.2023.2849

**Published:** 2024-05-22

**Authors:** Katherine McGann, Christopher N. Johnson, Michael Clinchy, Liana Y. Zanette, Calum X. Cunningham

**Affiliations:** ^1^ School of Natural Resources, University of Tasmania, Private Bag 55, Hobart, Tasmania 7001, Australia; ^2^ Department of Biology, Western University, London, Ontario N6A 5B7, Canada; ^3^ School of Environmental and Forest Sciences, College of the Environment, University of Washington, Seattle, WA 98195-2100, USA

**Keywords:** antipredator behaviour, ecology of fear, human impacts, perceived predation risk, predator–prey interactions, predator–prey naiveté

## Abstract

Recent experiments have demonstrated that carnivores and ungulates in Africa, Asia, Europe and North America fear the human ‘super predator’ far more than other predators. Australian mammals have been a focus of research on predator naiveté because it is suspected they show atypical antipredator responses. To experimentally test if mammals in Australia also most fear humans, we quantified the responses of four native marsupials (eastern grey kangaroo, Bennett’s wallaby, Tasmanian pademelon, common brushtail possum) and introduced fallow deer to playbacks of predator (human, dog, Tasmanian devil, wolf) or non-predator control (sheep) vocalizations. Native marsupials most feared the human ‘super predator’, fleeing humans 2.4 times more often than the next most frightening predator (dogs), and being most, and significantly, vigilant to humans. These results demonstrate that native marsupials are not naïve to the peril humans pose, substantially expanding the taxonomic and geographic scope of the growing experimental evidence that wildlife worldwide generally perceive humans as the planet’s most frightening predator. Introduced fallow deer fled humans, but not more than other predators, which we suggest may result from their being introduced. Our results point to both challenges concerning marsupial conservation and opportunities for exploiting fear of humans as a wildlife management tool.

## 1. Introduction

Recent experiments have demonstrated that the fear (antipredator behavioural responses [1]) that predators inspire in free-living wildlife can contribute substantially to the ecosystem-level impacts predators cause. Fear of predators can itself reduce prey population growth rates [2,3] and cause trophic cascades [1,4–8]. Fear of humans has also now been demonstrated to cause cascading impacts from carnivores to ungulates and rodents to plants in experiments simply broadcasting playbacks of people speaking [1,9–12]. New global surveys of the “unique ecology of human predators” [13, p. 858] show humans kill prey at much higher rates than other predators, warranting humans being termed a “super predator” [13, p. 858; 14,15]. Consistent with humanity’s unique lethality, a growing number of playback experiments have demonstrated that fear of humans far exceeds that of the non-human apex predator in the system. In Africa, 95% of carnivore and ungulate species tested (e.g. giraffe, leopards, hyena, zebra, rhinoceroses and elephants; *n* = 19 species) in Greater Kruger National Park ran more or fled faster on hearing humans compared to hearing lions, being on average twice as (2.0 times more) likely to run from humans than lions [16]. This effect size closely accords with the median likelihood of fleeing more or faster (2.1 times; range = 1.4–5.0) on hearing humans compared to hearing leopards, cougars, bears, dogs or wolves, demonstrated in comparable experiments on diverse carnivores and ungulates (cougars, European badgers, fallow deer, moose, white-tailed deer, wild boar) in Asia, Europe and North America [12,17–21].

Australia’s terrestrial mammal fauna has been called “the most distinctive in the world” given its preponderance of marsupials, presence of monotremes and high levels of endemism [22, p. 4351]. Human hunting of Australia’s native marsupials began with the arrival of Aboriginal peoples around 50 000 years ago and increased after European colonization [22]. Estimates of the current annual human predation rate on kangaroos and wallabies (*Macropus* spp.) accord with those of mammals worldwide [13]. Australia also has many introduced species [22,23]. Some of these, such as the six deer species now established in Australia, were introduced for the purpose of recreational hunting [24,25]. Native marsupials and introduced deer alike may thus be expected to most fear the human ‘super predator’, like mammals on other continents. That said, the distinctiveness of Australia’s terrestrial mammal fauna derives from Australia being the world’s largest island [22], and the antipredator behaviour of mammals on islands is often atypical due to processes such as founder effects [26–28]. Correspondingly, Australian mammals have been a focus of research on predator naiveté because it is suspected that they show atypical antipredator responses, ostensibly involving naïve, muted or absent responses to some predators [26–31]. Importantly, the relative fear of humans demonstrated by mammals in Australia remains to be experimentally tested.

To experimentally test whether mammals in Australia most fear the human ‘super predator’, we quantified fear responses [1] in four native marsupial species (eastern grey kangaroo, *Macropus giganteus;* Bennett’s wallaby, *Notamacropus rufogriseus*; Tasmanian pademelon, *Thylogale billardierii*; common brushtail possum, *Trichosurus vulpecula*) and introduced fallow deer (*Dama dama*), in Tasmania, using Automated Behavioural Response systems (ABRs; [32]) that video-recorded the reactions of animals to playbacks of the vocalizations of humans (women or men speaking calmly), dogs (C*anis lupus familiaris*; barking), Tasmanian devils (*Sarcophilus harrisii*; snarling), wolves (*Canis lupus lupus;* howling) or non-predator controls (sheep; bleating), following a well-established protocol [12,16,18,19]. Archaeology indicates all four marsupials have been hunted for millennia in Tasmania, with Bennett’s wallaby being hunted most [33,34]. At present, eastern grey kangaroos are ‘protected’ in Tasmania, having been hunted to near extinction by the 1950s for human consumption and dog meat, although thousands are still shot annually for crop protection [35,36]. Commercial and sport hunting of the other marsupials is permitted, along with shooting for crop protection, and at least 1 million are killed each year [37–39]. Over one-third of the fallow deer population is killed by hunters in some years [25]. Dogs are used by hunters to flush wallabies, pademelons and possums, but this is prohibited when hunting deer [37,40]. Tasmanian devils are the largest extant native predator and can kill Bennett’s wallabies, pademelons and possums, and kangaroo joeys and fawns [41–43]. While there never have been wolves in Tasmania, we included them primarily to test the responses of the fallow deer, because elsewhere fallow and other deer respond to wolf playbacks even where wolves have been locally extirpated [12,19,44]. A review of hundreds of predator playback experiments reported that prey respond significantly less to playbacks of novel predators (no ecological or evolutionary history), except when the novel predator’s vocalizations resemble a known predator’s, as with wolves and dogs [44]. For this latter reason, native marsupials might react fearfully to hearing wolves if they perceive them to be dogs [44].

Establishing the global pervasiveness of wildlife’s paramount fear of humans is directly relevant to conservation and management [1,16]. If paramount fear of humans pervades the planet, this presents both new challenges, given it is now clear that fear itself can significantly reduce wildlife numbers [1–3], and new opportunities, since manipulating fear of humans ought to thus provide one of the most potent and universally effective of all wildlife deterrents [12,16,45,46]. Playbacks of humans have recently been experimentally demonstrated to reduce crop damage by deer [12] and deter rhinoceroses from areas where they are at most risk from poaching [16,46], respectively reducing the impact of ‘problem’ wildlife and helping protect an endangered species. Predator playbacks are currently being considered to aid crop protection and reduce damage to native and endemic plants in Tasmania [25], and an additional aim of our experiment is to inform how this can most effectively be implemented.

## 2. Material and methods

### (a) Study area, species and sites

We conducted our experiment at Beaufront, a sheep grazing property in the Tasmanian Midlands (42°02′22.7″ S 147°35′25.4″ E), from July to October 2020. The five species whose responses we quantified (eastern grey kangaroo, Bennett’s wallaby, Tasmanian pademelon, common brushtail possum and fallow deer) are the most common wild herbivores on the property. All are shot for crop protection or hunted for sport on the property and the experiment was conducted during the March–November open season on deer. Humans, dogs, Tasmanian devils and sheep are all present on the property and were observed during the study (our ABRs occasionally recorded Tasmanian devils). The playback treatments used (humans, dogs, Tasmanian devils, sheep and wolves) thus represent, with the exception of wolves, species whose vocalizations can be expected to be familiar to the wild herbivores in the experiment.

We quantified the responses of wild herbivores to the playback treatments at 13 sites (all separated >1 km), located in areas of mixed grassland and dry eucalypt forest or woodland. Because all five playback treatments were broadcast at each site (see below), each site represents a stand-alone replicate of the experiment [19,32], and the experiment was thus replicated 13 times (the maximum number of sites that fit on the property). The >1 km spacing between sites was selected to ensure that different individuals were sampled at the different sites, which was almost certain concerning wallabies, pademelons and possums, and also highly probable regarding kangaroos and deer because the home range sizes of the smaller species are all <10 ha [47,48], and based on telemetry studies in south-east Australia and New Zealand those of the kangaroos and fallow deer are likely <100 ha [49–51].

### (b) Experimental design and field procedures

All five playback treatments (humans, dogs, Tasmanian devils, wolves and non-predator controls [sheep]) were broadcast at each of the 13 sampling sites. One ABR, comprising a video-enabled camera trap linked to a playback speaker [32], was deployed per site. A playback was broadcast each time the ABR’s camera trap was triggered, beginning 3 s after video-recording commenced and continuing for 10 s. Previous ABR experiments have shown that a 3 s delay is ample to identify a change in behaviour [16,32,52,53]. To ensure the broadcast of treatments was balanced and randomized across the diel cycle, the treatment set to broadcast if the ABR was triggered changed every 16 min (i.e. between 00:00 and 00:16, e.g. wolves would be broadcast, between 00:16 and 00:32, e.g. sheep would, and so on) and each ABR was programmed to broadcast all five treatments in random order, and then to do so again in a different random order while avoiding broadcasting the same treatment for >16 min, and so on, until the whole 24 h cycle was completed ([16,19,52,53]; for programmed playlists see electronic supplementary material, table S1). Prior to use, we verified that each programmed sequence was free of potential order effects. We used 9–11 exemplars of each treatment [9,12,16–19]. Sound files originated from online audio and video databases, library archives and personal recordings by the researchers [12,17]. Dog exemplars included recordings of multiple breeds, e.g. Alsatians, Dobermans and hunting hounds [12]. Each time the ABR was triggered the above-described programmed sequence determined the treatment broadcast but which exemplar of that treatment was played was randomly selected [16,19,52,53]. Playbacks were standardized to a volume of 60 dB at 10 m so that they were audible, but not startling, to animals within the 15 m detection range of the camera’s motion sensor [16,19,52,53]. As in previous ABR experiments, we placed bait (5 kg of ‘Stock nuts’, Laucke Mills, Greenock, South Australia) 10 m in front of the ABR as an attractant to maximize the number of reactions captured and help ensure that the animal’s position meant the reaction was readily quantifiable [9,18–20,32].

At each site, we strapped the ABR’s camera to a tree at a height of 1.5 m and positioned the connected speaker 0.5 m directly above the camera. Next, we set the focal point of the camera such that it was aimed at the pile of bait 10 m in front. Following this, we clipped vegetation within a 15 m radius of the front of the camera to reduce false triggers and ensure 100% detection at 10 m [16,52].

### (c) Quantifying fear responses

To quantify fear we evaluated (i) whether animals fled in response to the playback [1,16] and (ii) the percentage of time they spent vigilant if they did not flee [52,53]; on their first exposure to a playback treatment in each ‘independent exposure bout’ (defined below). Fleeing is among the most straightforward behavioural measures of fear [1], and being readily recognizable in virtually all species [16,53], it has been used to gauge the relative fear of humans in all prior comparable experiments [9,12,16–21]. Vigilance, in contrast, is often difficult to compare between species [53] and it is not uncommon that prey that show strong differences in the likelihood of fleeing from different predators are similarly vigilant to all predators [20,52,53]. Vigilance can nonetheless be useful in revealing different levels of fear toward different predators in species with a low natural proclivity to flee [17,52,54].

As in prior ABR experiments, an ‘independent exposure bout’ was defined as being separated by >60 min since the species last heard that treatment at that site, which is more conservative than the >30 min used to define independent occurrences in most camera trap studies [12,16,19,52,53]. To be scored as fleeing or vigilant in response to the playback, the animal had to be visible both before and after the playback began and not already fleeing or vigilant [16,52,53]. We operationally defined fleeing as taking more than three consecutive rapid steps [16,53] in the quadrupeds (possum and deer), and as bounding with head up with the legs moving >one body length between bounds, in the bipeds (kangaroo, wallaby, pademelon). We operationally defined vigilance as standing in one place with head up and neck vertical in deer [52], and with body vertical in marsupials. If more than one individual of a species was in view, we quantified the responses of up to the nearest five individuals [16,52,53]. We ensured high interobserver reliability by preliminary testing among observers, and following standard experimental procedure, observers were blind to treatment (i.e. videos were muted) [16,53].

### (d) Statistical analyses

We used generalized linear mixed models (GLMMs) to analyse (i) the probability of fleeing using a binomial (yes or no) distribution (mgcv package in program R [55]); and (ii) the percentage of time vigilant using the beta distribution (glmmTMB package [56]). For both models, we included an interaction between treatment and species (including main effects) that allowed each species to respond differently to the treatments. We included random intercepts of species nested within site, and video ID, to respectively account for potential non-independence of responses at the same site and potential non-independence of multiple individuals observed in the same video. We used small-sample corrected Akaike Information Criterion (AICc) to compare the full model containing the interaction with a reduced model containing only additive effects of treatment and species. We conducted these analyses considering either native marsupials as a whole or each species separately, and respectively refer to ‘taxon’ (marsupials as a whole) or ‘species’ in reporting the relevant results.

To complement the above analyses, we also tabulated the number of independent first exposure videos in which the species fled or not, or if it did not flee, whether it was vigilant or not (electronic supplementary material, table S2), and then used these tabulated data to conduct straightforward contingency table tests (log-linear or Fisher’s two-tailed exact) of treatment effects or treatment by taxon (marsupials versus deer) or species interaction effects. Finally, to test whether humans were perceived as the most frightening predator by marsupials as a whole, and by each species separately, we conducted pairwise *post hoc* tests comparing humans versus controls and humans versus the next most frightening predator, using both the results from our GLMMs and contingency table tests (electronic supplementary material, table S3). All analyses, whether using GLMMS or contingency tables, provided closely similar answers, corroborating the robustness of our results (electronic supplementary material, tables S3–S5). In the main text, the *p* values reported regarding pairwise *post hoc* tests are those from the GLMMs and they are corrected for multiple comparisons.

## 3. Results

We recorded 684 independent first exposure videos across the 13 sites, comprising 101 videos of eastern grey kangaroos responding to the 5 playback treatments, 121 of fallow deer, 139 of Bennett’s wallabies, 147 of Tasmanian pademelons and 176 of common brushtail possums (electronic supplementary material, table S2).

**Figure 1.**
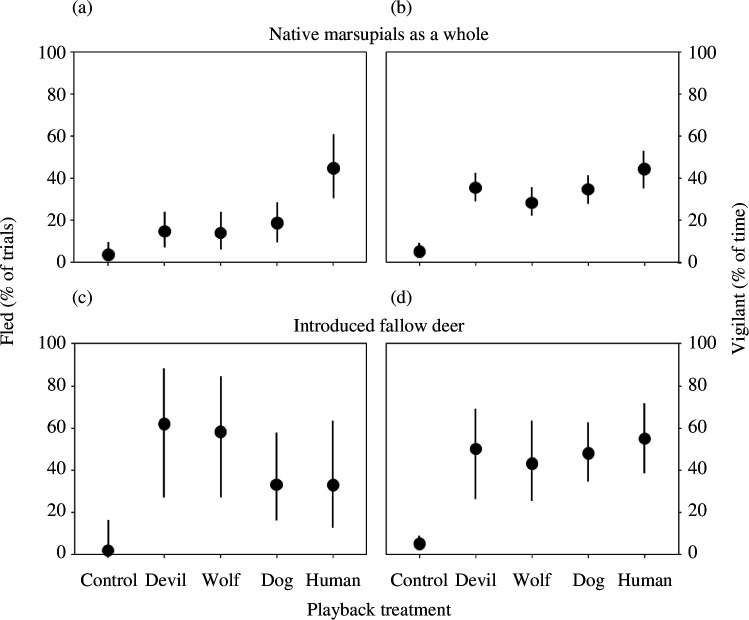
Effects of hearing playbacks of non-predator control (sheep), Tasmanian devil, wolf, dog or human vocalizations on fear responses (% fled, % of time vigilant) in native marsupials considered as a whole (*a,b*), and introduced fallow deer (*c*,*d*). A ‘trial’ means an ‘independent first exposure video’ as defined in the text. The percentage of time vigilant was only quantified in trials in which the species did not flee. Values are means ± 95% CI.

**Figure 2.**
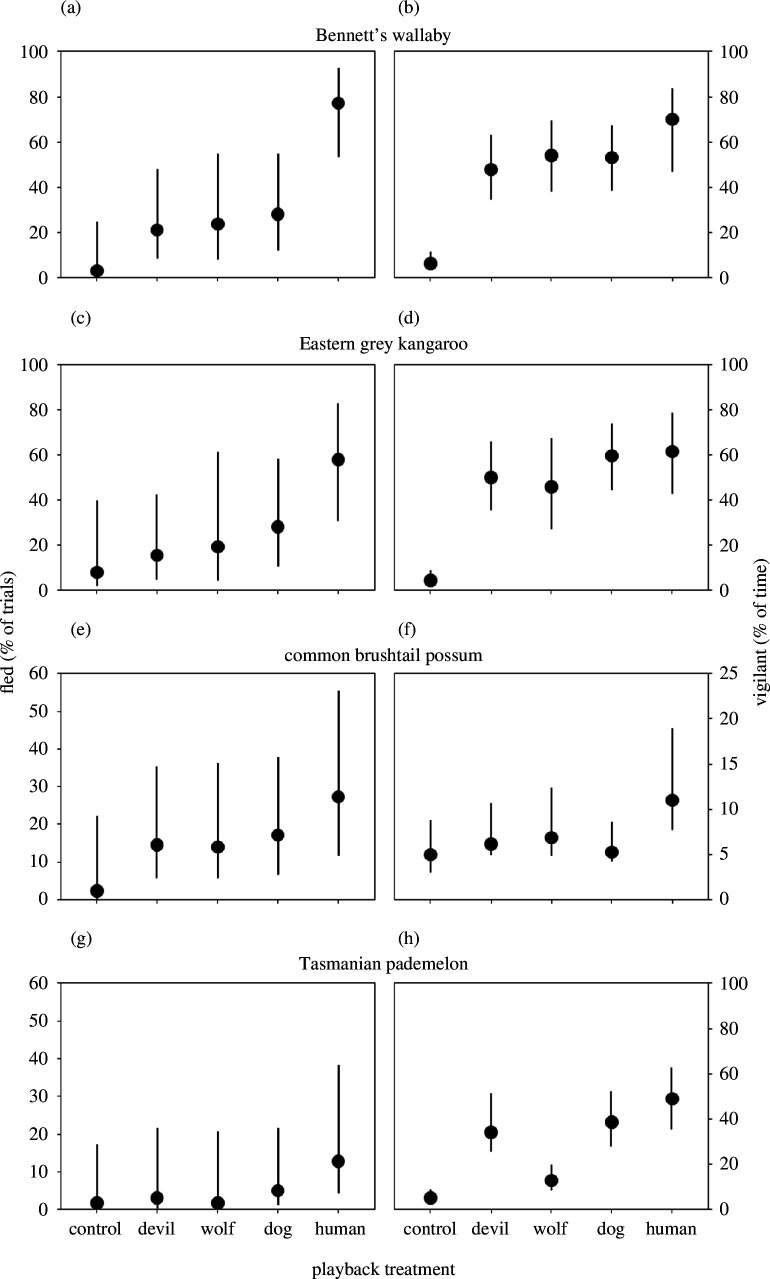
Effects of hearing playbacks of non-predator control (sheep), Tasmanian devil, wolf, dog or human vocalizations on fear responses (% fled, % of time vigilant) in Bennett’s wallabies (*a,b*), eastern grey kangaroos (*c*,*d*), common brushtail possums (*e*,*f*) and Tasmanian pademelons (*g*,*h*). A ‘trial’ means an ‘independent first exposure video’ as defined in the text. The percentage of time vigilant was only quantified in trials in which the species did not flee. Note the smaller scales on the *y*-axes*,* reflecting the lesser proclivity to flee in possums (*e*) and pademelons (*g*) and lesser proclivity to be vigilant in possums (*f*). Values are means ± 95% CI.

Native marsupials, considered as a whole (figure 1*a*), fled significantly more often upon hearing humans compared to hearing the next most frightening predator (dogs; *p* = 0.007, *n* = 256; electronic supplementary material, tables S2 and S3, videos S1 and S2), being more than twice as (2.4 times more) likely to flee from humans (44.3% versus 18.6% of trials). Considering fleeing in the four species of native marsupials separately, all demonstrated the same pattern of responses to the treatments, as each fled from humans roughly twice as often as the next most frightening predator, which in each case was dogs (figure 2), and all fled least from controls, with the result that there was no treatment by species interactive effect on fleeing (*p* = 0.932, *n* = 563; electronic supplementary material, tables S2, S4 and S5).

While all demonstrated the same pattern, the four marsupial species differed in their proclivities to flee upon hearing predators (figure 2). Wallabies were the most likely to flee from predators (38% on average), and like all the native marsupials they fled humans most (figure 2*a*; 77.2% of trials), 2.8 times more often than dogs (*p* = 0.004, *n* = 65; electronic supplementary material, tables S2 and S3, video S1) and 26 times more often than controls (*p* < 0.001). Kangaroos fled somewhat less from predators (30% on average), fleeing humans (57.7% of trials) 2.1 times more often than dogs (figure 2*c*; *p* = 0.090, *n* = 52; electronic supplementary material, video S2) and 7.2 times more than controls (*p* = 0.007). Possums fled even less from predators (18% on average), but nonetheless fled from humans (27.3% of trials) 11.4 times more often than controls (figure 2*e*; *p* = 0.044, *n* = 55) and 1.6 times more often than dogs. Pademelons rarely ever fled from predators (6% on average), so despite fleeing humans (12.8% of trials) 2.6 times more than dogs (figure 2*g*) and 7.1 times more than controls, these large relative differences were not statistically significant given the small number of times they fled. Pademelons that did not flee did spend significantly more time vigilant on hearing humans compared to controls (figure 2*h*; *p* < 0.001; electronic supplementary materials, tables S2 and S3), as did the other marsupials (wallabies, figure 2*b*, *p* < 0.001; kangaroos, figure 2*d*, *p* < 0.001; possums, figure 2*f*, *p* = 0.049), and all were most vigilant to humans (figure 1*b* and figure 2). Possums spent 1.6-fold more time vigilant to humans than to any other predator, but as they were apparently rarely vigilant to predators (figure 2*f*; 7% on average), this was not statistically significant (electronic supplementary materials, tables S2 and S3). Which non-human predator elicited the next-most vigilance after humans, and how much, varied among the marsupials (figure 2), resulting in there being a significant treatment by species interaction among them concerning vigilance (*p* = 0.029; electronic supplementary material, tables S4 and S5).

Comparing fleeing between the marsupials as a whole (figure 1*a*) and the introduced fallow deer (figure 1*c*), there was a significant treatment by taxon interaction (*p* = 0.040, *n* = 684; electronic supplementary material, tables S2, S4 and S5) due to the contrasting reactions to the predator treatments (*p* = 0.027, *n* = 575; considering predator treatments alone). In contrast to fleeing from humans significantly more often than the next most frightening predator, as native marsupials did (figure 1*a*), the deer were not statistically more likely to flee from one predator than another (figure 1*c*; *p* = 0.507, *n* = 42; electronic supplementary material, tables S2 and S3, video S3). Although the deer did not flee humans more than other predators, they did fear humans, as they fled humans significantly more often than controls (figure 1*c*; *p* = 0.029, *n* = 45), and those deer that did not flee spent significantly more time vigilant on hearing humans than controls (figure 1*d*; *p* < 0.001, *n* = 31). The deer were most vigilant to humans (figure 1*d*; 55% of post-playback time period), but only slightly, and not significantly more so than to other predators (range = 43–50%; *p* = 1.0, *n* = 20).

## 4. Discussion

Native marsupials in Australia, like carnivores and ungulates in Africa, Asia, Europe and North America [12,16–21], most feared the human ‘super predator’. The 2.4 times greater probability of fleeing more from humans than any other predator (figure 1*a*) and the consistency of fleeing most from humans throughout the marsupial community (figure 2), corresponds remarkably closely with the median probability of fleeing more or faster from humans (2.0 times) and consistency of this across communities, demonstrated in the previous comparable experiments on other continents [12,16–21]. Responses to dogs, as well as other predators, were tested in most of these previous experiments [12,17–20], including that in Africa [16], and the median greater likelihood of fleeing from humans versus dogs (2.5 times; range = 1.9–5.0) in these previous experiments corresponds even more strikingly with our results regarding the marsupials, which all fled second-most often from dogs. While the proclivity to flee varied among the four marsupial species, our vigilance results corroborate that all four species significantly fear humans (figure 2), just as supplementary measures corroborated the fear of humans in species with low proclivities to flee in the comparable experiment in Africa (e.g. elephants [16]). Our results demonstrating that marsupials in Australia most fear the human ‘super predator’ substantially expands the taxonomic and geographic scope of the growing experimental evidence that wildlife worldwide generally perceive humans as the planet’s most frightening predator [1,16].

Our results demonstrate that native marsupials in Australia are not naïve to the danger posed by the human ‘super predator’. As with the marsupials we tested in Tasmania, dogs are presently [16,20,35,37], or have been in the recent past [12,17–19], used to hunt the species included in most of the previous playback experiments testing fear of humans, which is why most have tested responses to dogs. Dogs are thus a threat, but as corroborated by the significantly greater fear of humans than dogs that our results demonstrate in correspondence with those from all prior comparable experiments, wildlife worldwide evidently recognize that it is humans who are the largest source of danger [13–16]. Previous playback experiments have concluded native marsupial herbivores in Australia are naïve to predator vocalizations [26,57] and predator playbacks will not deter native marsupials [58] because what was tested were responses to dogs, rather than the human ‘super predator’. The extant non-human apex predator in Tasmania, the Tasmanian devil, has recently been reported to inspire antipredator responses in wallabies, pademelons and possums [41,42]. Consistent with the previous experiments demonstrating greater fear of humans than non-human apex predators (e.g. lions, bears, wolves and leopards [12,16,17,19,21]), the native marsupials in our experiment were 3.1 times more likely to flee from humans than Tasmanian devils (figure 1*a*). While the larger, recently (<90 years) extinct Thylacine (*Thylacinus cynocephalus*) may arguably have been more effective at hunting these prey species than the Tasmanian devil [59], the low estimated number of Thylacines (pre-colonial population size of *ca* <4000 [60]) and archaeological and present evidence [33–39] suggest that humans have been and certainly now are far more lethal.

Australian marsupials have been argued to be predator naïve as a result of “relaxed predation” attributable to “the absence of large mammalian predators … in Australia during the past 40000–50 000 years” [31, p. 153]. However, there has been a large mammalian predator present in Australia for the past 50 000 years [22]; it is extremely dangerous [13,33,34]; our results demonstrate that all the marsupials fully recognize how dangerous it is and respond entirely appropriately and entirely consistent with how carnivores and ungulates on other continents respond to this predator; and that predator is of course—humans. This exemplifies what has long been a very common oversight in ecology, which is not recognizing or considering the role of humans as predators [1,13,16,61].

Fleeing involved abandoning the bait we provided (electronic supplementary material, videos S1 and S2) and thus entailed a foraging cost. While the lesser proclivity to flee of the smaller marsupials (possums and pademelons, figure 2*e* and *g*) may have been influenced by the relative benefit the bait represented, it has previously been shown that pademelons, as with many small ungulates on other continents [16,42,52,54], rely more on hiding than fleeing to evade predators, and possums being partly arboreal similarly rely more on climbing than fleeing [41]. Possums were apparently much less vigilant than the other marsupials (figure 2*f*), but this most probably reflects the challenge of comparing vigilance among species with different morphologies [16,53,54]. To be consistent with our operational definition concerning the other marsupials, possums were deemed to be vigilant when their whole body was upright and they were sitting on their haunches, which almost certainly requires more effort for a quadrupedal possum than a bipedal kangaroo, wallaby or pademelon.

That the introduced fallow deer did not flee from humans significantly more than other predators (figure 1*c*; electronic supplementary material, video S3) may be described as ‘atypical’, because this result: (i) significantly contrasts with the native marsupials (figure 1*a*); (ii) is contrary to experimental results regarding the reactions of fallow deer and other cervids in Europe and North America [12,19,20]; and (iii) differs from the pattern demonstrated by other wildlife worldwide [16–18,21]. It is also peculiar given how intensely fallow deer in Tasmania are hunted [25]. Atypical antipredator responses in mammals have been ascribed to processes such as founder effects [26–28]. The process of introducing a species may induce founder effects and a degree of semidomestication and there is ample genetic and other evidence that suggests fallow deer well exemplify this. Fallow deer have the greatest global distribution of any non-domestic large mammal [62], due entirely to repeated human introductions and translocations over millennia [63]. Fallow deer were introduced to England 900 years ago and kept in deer parks [63,64]. Just 12 individuals were introduced from England to Tasmania in 1829 and these and their progeny were kept in captivity for 20 years before release into the wild [62], after which numbers remained low for the next 130 years [25]. Fallow deer as a species have very low genetic variability due to this long history of introductions [63], and those in Tasmania show evidence of genetic bottlenecks occurring even after introduction [62]. Given the repeated founder effects and bottlenecks evident in their genetics, and history of intermittent captivity [62–64], the atypically muted fear of humans demonstrated by Tasmanian fallow deer appears consistent with current hypotheses concerning predator–prey naiveté [26–28].

Our results demonstrating that native marsupials in Australia most fear the human ‘super predator’ presents challenges for conservation, as fear of humans may itself adversely impact the population size of protected species [1–3,16], while offering opportunities, because exploiting their fear of humans ought to provide both an effective—and among the most potent—means of utilizing fear to help manage native marsupials in places where they are deemed overabundant [12,16,45,46]. Our results regarding the introduced deer suggest playbacks of humans can be expected to deter them [12], although no more or less effectively than playbacks of other predators. We recommend the use of human playbacks to aid crop protection and to reduce damage to native and endemic plants in Tasmania, because native marsupials and introduced deer co-occur in many areas [25] and the greater deterrent effect of hearing humans on the marsupials provides an advantage while not entailing any disadvantage in deterring deer. Introduced deer and other ungulates are causing adverse economic and environmental impacts worldwide [24,25,65]. We suggest there is a clear need for further experiments testing the fear that introduced ungulates have of humans and other predators, to determine whether, like most native species, most introduced ungulates also most fear the human ‘super predator’. Establishing how near fear of the human ‘super predator’ is to being all-pervasive is of practical significance, because it would obviously simplify utilizing fear in conservation and management—if one fear fits all [12,16,45,46].

## Data Availability

The datasets supporting this article have been uploaded as part of the electronic supplementary material [66].
